# Challenges for Adolescents With Congenital Heart Defects/Chronic Rheumatic Heart Disease and What They Need: Perspectives From Patients, Parents and Health Care Providers at the Institut Jantung Negara (National Heart Institute), Malaysia

**DOI:** 10.3389/fpsyg.2020.481176

**Published:** 2021-01-27

**Authors:** Sue Kiat Tye, Geetha Kandavello, Syarifah Azizah Wan Ahmadul Badwi, Hariyati Sharima Abdul Majid

**Affiliations:** ^1^Department of Psychology, International Islamic University Malaysia, Selayang, Malaysia; ^2^Institut Jantung Negara (National Heart Institute), Kuala Lumpur, Malaysia

**Keywords:** adolescents, congenital heart defects, chronic rheumatic heart disease, challenges, transition

## Abstract

**Objectives:**

This study aimed to describe the experiences and challenges faced by adolescents with moderate and severe congenital heart defects (CHD) or Chronic Rheumatic Heart Disease (CRHD) and to determine their needs in order to develop an Adolescent Transition Psychoeducational Program.

**Methods:**

The study involved seven adolescents with moderate to severe CHD/CRHD, six parents, and four health care providers in Institute Jantung Negara (National Heart Institute). Participants were invited for a semi-structured interview. Qualitative data were analyzed through the Atlas.ti 7 program using triangulation methods.

**Results/conclusions:**

We identified five themes concerning the experience and challenges of adolescents relating to: (1) emotional/psychological issues; (2) the progress of the illness; (3) relationship issues; (4) future preparation; and, (5) school and community. These themes were identified together with eleven subcategories. The staff expressed support for the development of the Adolescent Transition Psychoeducational Program and adolescents with CHD/CRHD and their parents were willing to participate in the program if their schedule allowed. Their suggestions to improve the program were classified into six categories, with two main themes, (1) the self-management of illness in life and the future; and, (2) social support. In conclusion, the findings from the situation analysis act as a basis for a conceptual framework that will contribute to the development of an Adolescent Transition Psychoeducational Program that aims to empower adolescents with CHD/CRHD, enabling them to manage challenges during the transition phase between childhood and adulthood.

## Introduction

The advancement of technology and research in congenital heart surgery has had positive effects on the survival rates of children with CHD, leading into adolescence and adulthood (Marelli et al., 2007; Stoutz and Leventhal, 2009). Living with CHD adds additional burdens to the life of an adolescent, particularly concerning medical symptoms and lifestyle (Brickner et al., 2000), mental and emotional challenges (Johnson, 2014; Wang et al., 2014), relationships (Dellafiore et al., 2016; Tye et al., 2017), employment (Pahl and Grady, 2012), and healthcare (Sable et al., 2011).

A number of patients with moderate to severe congenital heart defects usually require palliative or corrective surgery at an early age and require ongoing medical care (Marino et al., 2001). They face various complications and challenges, such as frequent readmission, neurocognitive deficits, restrictions in exercise, issues with pregnancy, and often need to take medication long-term to survive (Brummett et al., 1998; Schultz and Wernovsky, 2005).

Emotionally, if adolescents with these conditions fail to adapt to the changes resulting from an illness, it can cause anxiety, fear, and even increase their risk of depression (Brummett et al., 1998). Constant experiences of continuous negative feelings, especially hopelessness, during the lives of young people may jeopardize the risk of mortality in patients with cardiovascular diseases (Stern et al., 2001). Even though patients with CHD have a high risk of developing mood and anxiety disorders, their emotional health is often under-treated or ignored (Kovacs et al., 2009).

Socially, the illness might affect a patients’ relationship with their family, peers, and romantic partners. For instance, Pahl and Grady (2012) found that adolescents or young adults have difficulties in developing and maintaining significant relationships. Where interaction with peers is concerned, they often find themselves hindered by illness as they are unable to participate in social activities that require physical exertion such as hiking and camping, and subsequently feel left out and excluded by peers (Kendall et al., 2003).

Some patients experience overprotective and controlling behavior from their parents, which carried forward from childhood and persists into adolescence or even adulthood. This can cause a great barrier for adolescents in achieving their needs and developing autonomy as adults (Bertoletti et al., 2013). The impact of parental overprotection/control could be severe, as the risk of internalization behavior (anxiety and depression) and suicidal thinking can be high if the mother is overprotective, while the risk of externalizing behavior (aggression and rules breaking behavior) can increase if the father is over-controlling (Csomortani, 2011).

Similar to the normal population, adolescents or adults with CHD also desire an education or career (Pahl and Grady, 2012). The employment statistic of patients with disabilities and chronic illness conditions in Malaysia do not currently exist, but a study from the United States indicated that only 44% of respondents were employed and that 67% of those who were unemployed wished to work (Blomquist, 2006).

From the perspective of healthcare, adolescents with CHD are in a transition moment, when the services they receive shift from pediatric to adult management. This transition is often described as random and non-organized, which can cause non-adherence and difficult behavior. Moreover, if the transfer is sudden and unprepared, the adolescents might interpret the transition as a punishment and a sign of rejection (Viner, 2001). Adolescents with CHD are also expected to manage their health care, including taking medication and organizing appointments. Unfortunately, if these adolescents are not equipped with good analytical and decision-making skills and because emotion management skills are involved with risk-taking behaviors, they are more likely to adhere poorly to medical care, which may eventually lead to poor health outcomes (Pahl and Grady, 2012).

Patients with CRHD face challenges in relation to sociological and disease-specific factors as well as in terms of the health services they require. In the sociological domain, they can have life experiences of disease, poverty, powerlessness, and racism. The disease-specific domain comes from the fact that these patients have insufficient access to primary healthcare and lack information about their condition. Finally, patients with rheumatic heart disease often experience a lack of health communication and a positive experience is emphasized in the challenges of the health service domain (Haynes et al., 2020).

To address and resolve the issues above, particularly health care and lifestyle issues, various studies have suggested the need to have the services of formal Adolescent Transition to Adulthood programs to prevent discontinuity of care, financial or psychology related issues, to meet vocational, educational and other social needs and to promote future health (Hergenroeder, 2002; Moons et al., 2009; Sable et al., 2011; Kovacsa et al., 2012; Hays, 2015; Moceri et al., 2015; Said et al., 2015). However, Adolescent Transition Programs for CHD are not widely available (McPheeters et al., 2014). Even in the United States, only five percent of state mental health administrators have reported the existence of transition services or programs (Hunt and Sharma, 2013).

There is currently limited literature on the experiences and challenges faced by adolescents with CHD/CRHD in Malaysia. The challenges faced by adolescents in the Malaysian cultural context and the psychosocial programs needed to support them are still unclear. Based on the World Bank, Malaysia is categorized as an upper-middle-income economy (The World Bank, 2020). Compared with developed countries, patients in Malaysia face several challenges. First, only Kuala Lumpur, the capital of Malaysia, has a Congenital Heart Centre. Second, awareness of CHD and CRHD from the public is generally still low. Furthermore, to date, there is no proper transition program designed for CHD/CRHD in Malaysia. Our hospital planned to conduct this study to understand the challenges faced by adolescents with CHD/CRHD from the perspective of patients, parents, and healthcare providers, before designing a comprehensive transition program that includes education and psychosocial supports.

The purpose of this study is, (1) to describe the experiences and challenges faced by adolescents with moderate and severe CHD and CRHD, and (2), to explore what the precise needs of those patients are in our development of an Adolescent Transition Psychoeducational Program.

## Materials and Methods

This is a qualitative study that uses phenomenology to describe and analyze the common features participants have as they experience a phenomenon (Creswell, 2007). As the aim of the study was to identify the common themes/phenomena relating to the challenges faced by adolescents with CHD/CRHD, thus this research design was selected. Semi-structured interviews were conducted according to interview protocols, which were validated in a pilot study. The IJN Research Ethics Committee provided ethical approval for the research.

### Respondents

To align with the research objectives and design, purposive sampling was used to identify respondents who would provide information relevant to CHD/CRHD (Palinkas et al., 2013). The current study interviewed 17 respondents, seven adolescents with CHD/CRHD, six parents, and four health care providers. Data saturation and variability were achieved when no new themes were observed in patient’s challenges and needs from the program. Despite case numbers being low, the analysis was detailed and of quality.

The reason the researchers interviewed three groups of participants (patients, parents, and healthcare providers) was to ensure data trustworthiness by using the triangulation method of multiple respondents. This method ensured that fundamental biases arising from a single group are overcome to ensure the credibility of research findings. To ensure that the finding is valid, the researchers need to compare findings from different groups to determine whether they point to similar results (Creswell and Miller, 2000).

Adolescents with CHD/CRHD between the age of 16–19 years old at the date of recruitment and diagnosed with moderate or severe CHD or CRHD were included in the study. Patients with acquired heart diseases, genetic or mental disorders were excluded from the study. Patients who were home or bed-bound and unable to join the interviews were also excluded. The demographics of the participants are shown in Table 1. The interview sessions were conducted in the clinic and ward of the Institute Jantung Negara (National Heart Institute), Malaysia.

**TABLE 1:** Demographic of participants.

**Participants**	**Variable**	**Categories**	**Number**	**Percentage**
Patient	Gender	Male	4	57.14%
		Female	3	42.86%
	Ethnicity	Malay	5	71.42%
		Chinese	1	14.29%
		Indian	1	14.29%
	Diagnosis	Moderate	3	42.86%
		Complex	4	57.14%
Parents	Gender	Male	2	33.33%
		Female	4	66.67%
	Ethnicity	Malay	4	66.67%
		Indian	2	33.33%
Healthcare provider	Gender	Female	4	100%
	Ethnicity	Malay	3	75%
		Indian	1	25%

### Measures and Pilot Study

To ensure that the data collected are valid, the interview protocols were formed based on objectives and Creswell’s guidelines, including title and demographic, instructions, multiple similar questions for one theme, guidance for further elaboration, and verbal appreciation (Creswell, 2007).

First, three types of interview protocols were designed in English. Each set of interview questions had seven questions tailored to the perspectives and understanding of the three types of respondents. Example: “What has it been like living with your medical condition (Congenital Heart Defects)?”

The interview protocols were then sent to four translators for translation and back translation in Malay and Mandarin versions. Then, these interview protocols were sent to three experts for review to ensure that these questions were suitable for qualitative inquiry, participants’ educational and cultural context, achieving objectives, and fulfilling ethical and legal standards.

Finally, a pilot study was conducted on three adolescents with CHD/CRHD, three parents, and two health care providers to ensure their understanding of the interview protocols. The pilot study was recruited in the clinic. The findings from the pilot study showed that the participants were able to understand the interview protocols with some amendment in wording. No further questions were added or removed.

### Data Analysis

Moustakas’s (1994) phenomenological data analysis was adopted using the Atlas.ti version 8. First, the recorded interviews were transcribed and translated, followed by the identification of significant statements in the transcript. These statements would later be categorized into clusters to ensure they were sufficient to form a valid theme. To form a valid theme, we used the triangulation method of dividing multiple respondents into three groups (patients, family, and healthcare providers), in which they described similar shared concerns and overall experiences (Moustakas, 1994; Creswell, 2007).

## Results

### The Experience and Challenges Faced by Adolescents With CHD and CRHD

Initially, 41 categories of challenges were coded. Following the triangulation analysis for the three types of participants, only eleven categories were found valid, which was further classified into five themes as shown in Table 2. Ideally, all perspectives from the three different groups are shown in one category, based on the triangulation of multiple respondents. Due to word limits, only selected interview responses are presented below.

**TABLE 2:** Findings on the experiences and challenges of adolescents with CHD.

**Themes**	**Categories**
Challenges related to emotional/psychological issues	Feeling sad and afraid Feeling angry and denial
Challenges related to progress of illness	Experience physical symptoms Experience physical limitation/restriction Experience of lifestyles changes
Challenges related to relationship issues	Family interaction issue Friendship and social life issue
Challenges related to future preparation	Concern about their future/employment issues
Challenges related to school and community	Issue in schools Lack of support groups and community supports

#### Challenges Related to Emotional/Psychological Issue

##### Feeling sad and afraid

Emotional/psychological issues are one of the most common themes cited in interviews. Most adolescents reported a degree of sadness due to their illness. In some cases, they compared themselves with a healthy population. Others were worried about the progression of their illness. They were afraid that they may lose the capacity to do what they were able to do before. They became stressed because they were unable to manage their emotions

I scare about how long could I walk, the distance how long could I walk, I scare of it, I get stress then I can’t breath properly because of that.

(Patient, CRHD)

(Really difficult, if we feel he is sad… When he feels sad, isolated, and he wants to talk about it, he can’t, like isolating himself.)

Memang susah, jika kita rasa dia sedih, mungkin dia rasa sedih, dipinggirkan, tapi dia nak luah, tak boleh, macam mengasing dirinya.

(Parents, CHD)

##### Feeling angry and in denial

The experience of being a patient triggered feelings of anger and frustration in some patients. If these adolescents are unable to control their emotions, it affects their relationships, as well as their medical condition:

(Ya, because when the emotion is not stable and meet up others, I might release anger on them.)

Aha, sebab nanti kalau macam emosi sedang tidak stabil dan bila jumpa orang nanti, saya akan termarah mereka.

(Patient, CHD)

So that’s why we have a lot of compliance problem among the teenagers, because most of them they feel… I’m well, I don’t need to take medication, a lot of denial, a lot of anger, they always angry, angry with their family.

(Healthcare provider)

#### Challenges Related to Progress

##### Experience physical symptoms

Common physical symptoms experienced by adolescents with complex heart disease were tiredness and shortness of breath. Some complained of pain and dizziness. Due to the progression of their illness, some patients would need medication to control their symptoms. Some patients had significant symptoms like fainting, causing them to be fearful of recurrence and being a burden to others.

(Like…. Afraid I may suddenly faint. This will cause trouble to others, like calling an ambulance to this hospital. This condition will frighten other people)

Macam. Takut tiba-tiba saya pengsan, akan menyusahkan orang, macam panggil ambulan ke hospital ini. Menakutkan orang la macam keadaan ini.

(Patient, CHD)

(Sometimes when I looked at her difficult in breathing, I do not allow her to go to school.)

*Kadangkala dia macam susah nak bernafas, kadangkala fikir juga. Ah*… *Tengok dia kadangkala tak bagi dia pergi sekolah.*

(Parents, CHD)

##### Experience physical limitation

The majority of adolescents experience challenges due to physical limitations. Compared to the other categories, physical limitations ranked highest. Physical limitations could be caused by the progression of the illness, or due to unwarranted restriction by people around them. This physical limitation has a bigger impact on those who previously had normal functional capacity. These patients experience more grief, frustrations, and have difficulty dealing with their worsening functional capacity:

For example, my front gate in my school to behind gate is so long, must walk… so I stand and rest in three places and breath deeply, then only I can walk.

(Patient, CRHD)

(Ok, the other challenges I feel is from the perspective of sporting activities … Starting from the age of 13, I purposely limits her activities. Thankfully until 12 years of age, she manage to be active in sports. However, I feel this current restriction should not be a problem as there are still a lot of light activities she can join.)

*Ok, cabaran-cabaran lain itu saya berasa dalam segi active sukan itu memang dia. Bermula daripada 13 tahun memang dia*… *Saya memang had-kan dia punya activiti. So*… *dari kecil sampai 12 tahun, Alhamdulilah dia sempat bersukan, jadi saya berasa tak ada masalah kot, ada banyak lagi sukan yang ringan-ringan yang dia boleh join.*

(Parents, CHD)

##### Experience of lifestyles changes

As the illness progresses, adolescents with complex heart disease need to adapt and manage lifestyle changes, particularly those with mechanical valves. These types of patients need to take anti-coagulants throughout their life to prevent mechanical valve dysfunction. They need regular blood reviews, adjustments to their diet, and lifestyles. Furthermore, female patients of childbearing age have an increased risk of morbidity and mortality.

My medication. It was large different before 2 years and now.

(Patient, CRHD)

(I just need to prepare my child and myself… When using a mechanical valve, there are few things she is forced to comply with. First is the diet. Second… it might cause bleeding if she doesn’t follow her diet precautions. And third… the most challenging, because she is a girl, she is not encouraged to get pregnant.)

*Cuma saya perlu persiapkan saya dan anak saya*… *Apabila menggunakan injap mechanical, ada beberapa perkara yang dia paksa patuhi. Pertama permakanan. Kedua*… *boleh berlaku pendarahan bila-bila masa jika permakanan tadi dia tidak patuhi. Dan yang ketiga. agak. paling mencabar, because dia adalah perempuan, dia tidak digalakkan untuk pregnant.*

(Parent, CRHD)

#### Challenges Related to Relationship Issues

##### Family interaction issue

There were controversial responses from the interviewees with regards to family interactions. Four patients did not have any family issues. Others reported issues like overprotectiveness, pampering, and a lack of independence. This could be partly due to a family’s fear of losing the child. Some interviewees felt that this reliance on parents was undesirable as it could limit an adolescent person’s capacity to care for themselves, especially in the absence of the parents.

And the another thing is … ya… my family doesn’t like me to go out more than my other siblings. So it’s like they kept me in house, doesn’t let me to get social.

(Patient, CRHD)

(Sometimes I am afraid of even letting her take the bus alone. I am frighten. Usually I will drive her wherever she wants to go. However, I am aware that at some point, I have to let go as she cannot stay with me forever right? She will need to make friends and go dating. I have thought a lot about that, and I worry about what I should do.)

Kadangkala nak lepas dia naik bus pun takut! Takut kan? Selalu saya drive kereta, mana dia nak pergi pun saya bawa. Bila satu tahap tak kan saya nak bagi dia duduk dengan saya sahaja kan? Dia akan berkawan, dia mesti nak dating pada masa akan datang. Banyak terfikir, masa itu la saya risau apa nak buat.

(Parents, CHD)

##### Friendship and social life issue

Despite sufficient data for this category to form a valid code, the majority of the participants alleged that they did not have any issues in forming friendships. Some adolescents did, however, need to frequently explain their illness to their friends to prevent misconceptions and unintentional injuries. Those unable to manage their emotions were at times treated with hostility and isolated by peers and some were subjected to bullying.

So far my life with this disease is quite difficult, quite challenging. There is a lot of things I need to manage, there is a lot of things I need to adjust, there is a lot of things I need to explain. And that happened everywhere, high school, university and within my friends.

(Patient, CRHD)

Because once they feel frustrated, they will become… err… either they will isolate themselves and start doing their own things since they cannot join their peers. This may cause some grief among the family… the parents and their children or it can cause lots of animosity against the teachers or friends. Sometimes they are subjected to bullies in the school.

(Health care provider)

#### Challenges Related to Future Preparation

##### Concern about their future/employment issues

Three adolescents and one parent discussed that they did not think about the future. Another parent remained positive about the future. However, the majority of respondents were concerned about the future, especially employment. Patients with complex heart disease may be physically restricted, leading to stereotypes and discrimination that they are unable to perform tasks.

Because industrial company will want healthy and fit employees right? So I think it will be a challenges for me to get a job.

(Patient, CRHD)

First of all is employment. They will have problem in employment. Of course some of the adolescents dropped of their school because their illness. Financial they are going to have financial issue, they need financial support. If they are capable enough to work and providing, sometimes if the disease itself progress, they might not able to work. Sometimes when these adolescents, when they proceed into adulthood, they might need to support their parents. So I foresee a lot of financial issue.

(Health care provider)

#### Challenges Related to School and Community

##### Issue in schools

The issues faced in schools range from minor to major. Four out of the seventeen participants claimed that they coped well in school, but some had issues related to acceptance by teachers and peers, a lack of understanding of their disease, high absenteeism, and poor school performance.

(The teacher and my peers have difficulty accommodating me, I am less favored because I seldom go to school due to chest pain and this is unavoidable. I am unable to achieve the target given by my teacher as I did not go to school. So, it is difficult…)

Penerimaan orang terhadap diri kita agak susah, sebab sekarang di alam persekolahan pun dah macam, orang tak suka sangat sebab saya jarang datang sekolah, sebab sakit dada dan tak boleh dielakkan, so macam cikgu bagi target tak tercapai sebab kita tak datang ke sekolah. So susahlah.

(Patient)

Sometimes even when they are undergone surgery and considered sort of cured from the disease, they are also a lot of hesitancy in the part of teachers/school to allow them to take part in physical activities or take part in any activities in school, this makes them feel alienated and frustrated.

(Health care provider)

##### Lack of support groups and community supports

The health care providers acknowledged that providing medical care and education alone was not sufficient to help this group of patients. Unfortunately, there is a lack of adequate support groups and community support for adolescents with heart disease.

(Difficult, because we Kelantanese don’t have any association. Before have heart foundation, but now when we find it, it doesn’t exist.)

Susah, sebab kita Kelate tak ada apa-apa kesatuan. Dulu ada Yayasan jantung, tapi kita cari-cari tak ada.

(Parents, CHD)

Even Down Syndrome they have Kiwanis. Argh… Congenital Heart Defects, they don’t. Even the cancer, the children with cancer, they have their own supports, err… what else do I know? Cancer especially is very strong, but congenital heart defects. We don’t.

(Health care provider)

### The Needs of the Program Development

To explore what the patients might need from a new support program, the researchers first reviewed the staff perception of an Adolescent Transition Psycho Educational Program and the willingness of adolescents with CHD and their parents to participate in such a program. Second, important elements of the program were suggested by the respondents.

All of the health care providers who participated in the interview felt that the Adolescent Transition Psycho Educational Program would be helpful and were happy to support the program. Both the adolescents with CHD and their parents showed a positive attitude toward the program. All adolescent respondents were keen to join the program, but two of them said they would need to review their schedules before committing to it. Some parents also had encouraging responses, pending 50% (*n* = 3) need to review their schedules.

Atlas.ti version 8 was used to review the elements of the program and recognized 19 categories. After triangulation of the three types of respondents, four valid categories were identified, which have been classified into two themes, as indicated in Table 3.

**TABLE 3:** Findings on the elements to be included to the program.

**Themes**	**Categories**
Coping with illness and future	Self-management on illness and emotions Preparation for future
Social support	Support group Psychosocial support

#### Self-Management of Illness and Life

##### Coping with illness and emotions

Some adolescents felt they had difficulties in handling their illness and hoped the program would help them cope with the challenges associated with the illness. Parents and health care providers hoped the program would help the adolescents self-manage health issues and become more independent.

(I don’t know what specifically hospital can offer, but give anything that can help us cope with the illness.)

Saya tak tahu specific apa hospital boleh bagi, tapi bagilah apa yang boleh bantu saya menangani penyakit ini la.

(Patient, CHD)

(What is suitable, what is not suitable. But when she grow up, I felt I need to give her a chance to take care of herself.)

Apa yang sesuai, apa yang tak sesuai. Tapi bila dah dewasa nanti, saya rasa saya kena bagi peluang untuk dia menguruskan diri sendiri.

(Parents, CHD)

##### Preparation for future

Some respondents were uncertain about the future of these adolescents, especially with regards to the progression of illness and employment issues. They outlined that they would like the program to provide adolescents with guidance for preparing and dealing with potential issues in the future:

(Like if my situation deteriorate at the future, what I should do? Do I need to do operation again?)

Macam jika situasi saya teruk pada masa depan, bagaimana saya perlu buat? Perlu saya buat pembedahan lagi ke?

(Patient, CRHD)

(I … what the best is… I also don’t have information from hospital la. What my child can do to… Move forward and pursue her career.)

*Saya*… *apa yang terbaik adalah*… *saya pun tak tahu daripada hospital la. Apa yang anak saya boleh buat untuk*… *maju jaya dan mengejar kerjaya nya.*

(Parents, CHD)

#### Social Supports

##### Support groups

A number of respondents suggested that having a patient support group might be helpful. They outlined that it would reassure them that they are not alone and that others share similar concerns. It would also enable them to learn constructive coping strategies from one another.

Maybe for the feelings, maybe we can have the support group or regular meetings with the counselors.

(Patient, CRHD)

Support group I think is very important because they can link, they can do activities together, they don’t feel they are alone. So they can have their own organization. like a group, so that they know. someone from the world, they have the same situation. condition with them.

(Healthcare provider)

##### Support from psychologists/counselors

The respondents outlined that they wanted support from psychologists and counselors, who could assist them in managing the challenges they face, as discussed by ACHD9 and HCP4:

(Probably the hospital can set up a counseling department, then for those who don’t know what to do, the counselors could tell them how to manage.)

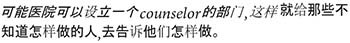

(Patient, CHD)

Yes, psychological program, how to cope with the disease. I am not sure whether we can put some elements into their program.

(Healthcare provider)

## Discussion

The findings of the current research concurred with past literature on the topic. It has been reported that adolescents with CHD experience more emotional issues than those without chronic disease (Johnson, 2014). There is a higher prevalence of anxiety and depression (Wang et al., 2014), and a higher risk of internalizing and externalizing problems (Karsdorp et al., 2007) in adolescents with a chronic disease. Physical limitations and restrictions were the most common issues reported in the present study. This increased the risk for lower quality of life (Dahan-Oliel et al., 2011; Riaz et al., 2018) and worsened psychosocial issues (Johnson, 2014).

Moreover, data in the current study showed that the respondents hoped that a program would help guide adolescents with CHD/CRHD, enabling them to self-manage their illness, and preparing them for facing possible challenges in the future. These findings are validated by Chen et al. (2016), whose study observed that it was crucial to assist adolescents with CHD and help them to learn health self-management and how to cultivate a positive attitude when handling disease (Chen et al., 2016, 2019).

With better knowledge and understanding of their cardiac condition adolescents with CHD are expected to have a better health-related quality of life (Wang et al., 2014). Even though some adolescents were aware that they needed to take active responsibility for their health, they did not know where to begin. Moreover, they were not certain about their lifespan due to the progress of the illness and were unsure of how to manage it. In the Malaysian cultural context, adolescents outlined that medical staff tended to communicate only with caregivers and expected the caregivers to deliver the message to the patients, instead of directly communicating with the patients. Thus, adolescents with CHD/CRHD were not sure of the progress of their illness, and only followed the instructions of the parents and medical staff in taking care of themselves.

Chen et al. (2016) have suggested that a transitional health passport and clinical interventions to smoothen the transition process from adolescence to adulthood should be developed. Lee et al. (2014) also suggest that a comprehensive educational program and support group that enhances the task-oriented coping-mechanisms of adolescents will further increase their resiliency toward illness. Transitional health-related issues, such as medical-related issues, employability, and sexuality, should be targeted in such a program to prepare adolescents for their future (Canobbio, 2001).

Psychosocial supports and support groups are another theme or element that could be included in a support program. Psychosocial support from counselors could provide psychological interventions. Support groups provide a place where patients who have similar conditions can gather and meet, enabling them to understand, learn, and support one another.

The patients included in this study expressed emotional challenges. These findings are supported by another recent study by Sheikh et al. (2019), stating that most patients with CRHD suffer from moderate levels of anxiety and depression, which are associated with physical pain, social functioning, and general health. In comparison to the general population, patients with CHD showed a higher prevalence of depression, anxiety, and posttraumatic stress disorder, which lead to a greater risk for cardiovascular morbidity and mortality. On top of that, only a small number of people receive psychosocial treatment (Andonian et al., 2018).

Another contributing factor could be avoidance or disengagement coping strategies, such as maladaptive coping, as it is correlated with high levels of anxiety, depression, and somatic complaints (Compas et al., 2006). Even though avoidance coping could contribute to resiliency in managing stressful events, findings have shown that task-oriented and emotion-oriented coping could cultivate better resiliency for adolescents with CHD (Lee et al., 2014). Unfortunately, a worrying tendency seen in CHD patients, is that these patients tend to use avoidance strategies, such as denial or distract themselves with something unrelated, more often compared with a normal population (Iwaszczuk et al., 2014).

Support groups for patients with CHD are still underdeveloped in Malaysia. NGOs have provided support for patients with terminal diseases like cancer, but patients with CHD still lack support groups and some can only attend support groups overseas through internet access. However, the use of online support is not yet a common practice.

Patients in Malaysia have similar needs to those in other countries. They would like to receive an education and improve their self-management of their condition through knowledge and by receiving psychosocial support. Additional challenges faced by patients in Malaysia compared with developed countries include the lack of congenital heart centers (so far only one is available in Kuala Lumpur), a lack of awareness of CHD/CRHD, and the absence of an Adolescent Transition Program for patients who require complex congenital heart surgery in government hospitals, particularly in Sabah and Sarawak, where they need to join a waiting list to transfer to Kuala Lumpur for surgery.

### Strengths and Limitations

A key strength of this study is its triangulation of multiple respondents (patients, parents, and health-care providers) to ensure reliable data. It compared multiple sources to determine whether they point to similar results and to avoid fundamental biases from a single group.

Even though the saturation of data was achieved, the study is limited by its small sample size. There is also a potential selection bias as we used purposive sampling to recruit respondents in the study. In Malaysia, the majority population has Malay, Chinese, and/or Indian heritage, and there is a lack of representation from other minorities, such as Kadazan and Ibans. Moreover, there is an absence of representatives from states, such as Sabah and Sarawak. Furthermore, patients with comorbid conditions (genetic disorders and mental disorders) were excluded from the study. The present study highlighted the needs of patients, but there was a lack of comprehensive comparison with international data like the research of APPROACH-IS.

## Conclusion

The present study suggests that an Adolescent Transition Psycho educational Program should be developed to assist adolescents with moderate to severe heart disease. This program would assist them to develop tools for self-managing emotional and psychological issues, the lifestyle impact of the disease progression, and prepare them for the future. Support groups and psychosocial support should also be incorporated into the program.

## Data Availability Statement

The datasets generated for this study are not publicly available. Requests to access the datasets should be directed to windofheart2000@msn.com.

## Ethics Statement

The studies involving human participants were reviewed and approved by IJN Research Ethics Committee. Written informed consent to participate in this study was provided by the participants’ legal guardian/next of kin. Written informed consent was obtained from the individual(s), and minor(s)’ legal guardian/next of kin, for the publication of any potentially identifiable images or data included in this article.

## Author Contributions

All authors listed have made a substantial, direct and intellectual contribution to the work, and approved it for publication.

## Conflict of Interest

The authors declare that the research was conducted in the absence of any commercial or financial relationships that could be construed as a potential conflict of interest.
